# Multiple Loci Are Associated with Dilated Cardiomyopathy in Irish Wolfhounds

**DOI:** 10.1371/journal.pone.0036691

**Published:** 2012-06-25

**Authors:** Ute Philipp, Andrea Vollmar, Jens Häggström, Anne Thomas, Ottmar Distl

**Affiliations:** 1 Institute for Animal Breeding and Genetics, University of Veterinary Medicine Hannover, Hannover, Germany; 2 Veterinary Clinic for Small Animals, Wissen, Germany; 3 Department of Clinical Sciences, Faculty of Veterinary Medicine and Animal Science, Swedish University of Agricultural Sciences, Uppsala, Sweden; 4 ANTAGENE, Animal Genetics Laboratory, Limonest, France; Pennsylvania State University, United States of America

## Abstract

Dilated cardiomyopathy (DCM) is a highly prevalent and often lethal disease in Irish wolfhounds. Complex segregation analysis indicated different loci involved in pathogenesis. Linear fixed and mixed models were used for the genome-wide association study. Using 106 DCM cases and 84 controls we identified one SNP significantly associated with DCM on CFA37 and five SNPs suggestively associated with DCM on CFA1, 10, 15, 21 and 17. On CFA37 *MOGAT1* and *ACSL3* two enzymes of the lipid metabolism were located near the identified SNP.

## Introduction

Dilated cardiomyopathy (DCM) is a myocardial disorder which affects dogs as well as humans. The disease is characterized by systolic dysfunction caused by an impaired myocardial contractility and progressive dilation of the left or both ventricles. Affected dogs often develop signs of congestive heart failure (CHF) during the progession of the disease or die from sudden cardiac death. A familial background for DCM has been suggested in several dog breeds and in people [1]–[3].

In humans, DCM is a genetically heterogenic disease [4], [5]. Rare variants of genes encoding predominantly sarcomeric, cytoskeletal or nuclear proteins have been shown to account for monogenic familial forms of DCM [6]. Until now, mutations in more than 30 genes have been associated with DCM [7]. There is, however, also some evidence that common genetic variants might play a role for causing DCM in humans [8].

Several human candidate genes have been ruled out as causative in Doberman Pinschers, Irish wolfhounds and Newfoundland dogs [9]–[18].

Recently, using mainly a linkage, candidate gene or genome-wide association analyses evidence was found for genomic loci being associated with cardiac diseases in dogs. In Boxer dogs from US, a mutation in the striatin gene was found associated with arrhythmogenic right ventricular cardiomyopathy (ARVC) but could not explain all cases of ARVC [19]. In Portuguese waterdogs, for a genomic region spanning 3.9 Mb on dog chromosome 8 linkage has been demonstrated for a juvenile form of DCM [20].

A mutation in the *ACTN2* gene has been associated with DCM in some, but not all, affected Doberman Pinschers [21]. In addition, it has been shown that a locus on canine chromosome (CFA) 5 was associated with DCM in Doberman Pinschers explaining about 50 percent of DCM cases in dogs from Germany and the United Kingdom [22].

In Irish wolfhounds, the incidence of DCM reaches approximately 20 percent [23] which leads to the highest cause-specific mortality rate for cardiac disease in the breed compared to all other breeds [24]. The mean age of onset has been estimated at 4.52±2.0 years and female dogs are less frequently affected and develop the disease at an older age than males [23]–[25]. This suggests a protective effect in female Irish wolfhounds. A major gene model with sex-specific allele effects was the most plausible explanation for the inheritance of DCM in Irish wolfhounds whereas a monogenic mode of inheritance of DCM had been rejected using complex segregation analysis [26]. Echocardiographic reference values have been established for Irish wolfhounds, facilitating the diagnosis of DCM in this breed [27], [28]. The objective of this study was to identify loci associated with DCM in Irish wolfhounds. Therefore, we conducted a genome-wide association study (GWAS) to identify susceptibility loci for DCM in Irish wolfhounds.

## Results

### Mapping Genomic Regions

**Figure 1:**
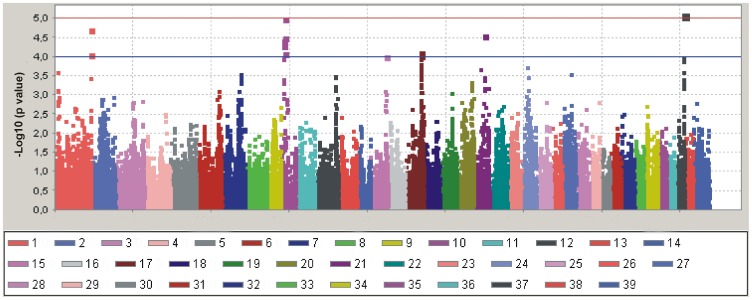
Manhattan plot of the genome-wide association study for dilated cardiomyopathy in Irish wolfhounds from Europe using a general linear model analysis. The genome-wide p-values (–log10 p-values) for the SNP effect are plotted against marker position on each chromosome. X-axis indicates marker number. Chromosomes are differentiated by colours. Colours are given below the plot. Red line indicates threshold value of probabilit*y* for significant association with DCM. Blue line indicates threshold value of probability for suggestive association with DCM.

A genome-wide association study was performed for 106 Irish wolfhound-DCM-cases and 84 Irish wolfhound-DCM-controls using the canine Illumina high density (HD) beadchip (Illumina, San Diego, CA, USA). The 190 Irish wolfhound samples (Table S1) were collected in Central Europe (including samples from Germany, The Netherlands and Belgium), France and Sweden (including samples from Denmark and Norway). Using general linear model analysis (GLM) with sex, inbreeding coefficient and the first three principal components as covariates a significant association with DCM was identified on dog chromosome 37 (Figure 1, Table 1). The corresponding quantile-quantile (Q-Q) plot illustrates the level of potential p-value inflation (Figure S1). The error probability threshold was 9.72×10^−6^ for DCM using Bonferroni correction at a p-value of 0.05 for 5142 independent tests. The assumption of 5142 independent tests was based on pair-wise correlation coefficients among alleles for all SNPs used in GWAS and threshold for r^2^<0.2. In order to provide moderate evidence of association a threshold of 1×10^−4^ was used [29], [30]. Applying this less stringent threshold resulted in five additional SNPs associated with DCM on CFA1, 10, 15, 17 and 21 (Figure 1, Table 1).

**Table 1:** Summary of results for the genome-wide association study using a mixed model analysis for dilated cardiomyopathy in European Irish wolfhounds.

Locus	Position (bp)	SNP	Gene	Major	Minor	MAF	MAF	MAF	OR	OR-L	OR-U	-log10P	-log10P
CFA				allele	allele	all	AFF	UNAFF				GLM	MLM
1	123,630,555	BICF2P937484	–	C	T	0.12	0.08	0.18	2.48	1.32	4.67	4.72	3.70
10	24,159,608	BICF2P801304	*ARHGAP8*	A	C	0.06	0.03	0.11	3.62	1.46	9.01	4.51	3.56
15	61,260,406	BICF2S23319191	*FSTL5*	C	A	0.26	0.22	0.32	1.63	1.03	2.59	4.02	3.64
17	58,604,566	BICF2S23232515	–	C	T	0.48	0.52	0.41	1.56	1.03	2.36	4.12	3.59
21	40,670,543	BICF2P5199741	*PDE3B*	G	A	0.29	0.32	0.26	1.31	1.20	2.06	4.57	4.08
37	31,801,266	BICF2G630134189	–	G	A	0.43	0.37	0.51	1.73	1.14	2.61	5.10^*^	4.14

Identified loci on dog chromosome (CFA), position in base pair (bp), SNP identification and localization, gene name (Gene) for intragenic SNPs, major and minor allele, the minor allele frequency (MAF) in all dogs, affected and unaffected dogs (MAF all, aff, unaff) odds ratios (OR) with lower and upper 95% confidence limits (-L, -U), −log10 p-value of a general linear model (GLM) and mixed model analysis (MMA) is given.

Mixed model analysis (MLM) including sex, inbreeding coefficient and the first three principal components as covariates confirmed the GLM analysis (Figure S2). The SNP highest associated with DCM on CFA37 was identical in both analyses. Due to fact that we included dogs of several countries the possible impact of population stratification on the GWAS result was evaluated for the first three principal components in scatter plots (Figure S3). The results indicated that stratification probably did not impair the association results.

In order to quantify the impact of the single SNPs on occurrence of DCM the odds ratios (OR) were estimated. The ORs for the single SNPs were between 1.31 for BICF2P5199741 on CFA21 and 3.62 for BICF2P801304 on CFA10.

The highest associated SNP BICF2G630134189 on CFA37 is located about 40 kb distal to *MOGAT1* and 80 kb proximal to *ACSL3.* The SNPs on CFA10, 15 and 21 are located in the genes *ARGHAP8*, *FSTL5* and *PDE3B*, respectively (Table 1) while the other two SNPs moderately associated with DCM are located in intergenic regions (Figure S4, S5, S6, S7, S8, S9).

To identify haplotypes associated with DCM linkage disequilibrium and haplotype block analysis was performed. Haplotype blocks containing the SNPs associated with DCM were identified for five chromosomes (Table S2). On CFA21, no significantly associated hapotype block (p-value <0.05) was observed. The size of the associated haplotype blocks varied from 78 kb on CFA15 to 794 kb on CFA17. On CFA17, the genes *SPAG17* and *TBX15* are within the associated haplotype block. On CFA37, the associated haplotype block of 294 kb contains the genes *MOGAT1* and *ACSL3*.

## Discussion

The genome-wide association study in Irish wolfhounds demonstrated the potential involvement of six loci in DCM. We identified one SNP significantly associated with DCM on CFA37 and five SNPs with evidence of association with DCM on CFA1, 10, 15, 17 and 21. The same SNPs were consistently mapped using GLM and MLM analyses. Our results suggest that DCM in Irish wolfhounds is not inherited as a simple Mendelian trait. This finding is in agreement with a previous complex segregation analysis which found an autosomal dominant major gene model with sex-specific allelic effects as the best suited inheritance model for DCM in Irish Wolfhounds [26]. An oligogenic inheritance of DCM might be explained by the fact that dogs from several breeds including Great Danes, Scottish deerhounds, Barsoi and Tibet mastiffs were crossed with few remaining Irish wolfhounds to conserve this breed, which at the time, was close to extinction. This admixture may have lead to many gene variants contributing to development of DCM in Irish wolfhound population. This is in contrast to dog breeds with pure bred founders which are classified as closed populations and might harbour only a limited number of disease causing alleles.

For association study we included Irish wolfhounds originated from several European countries. Nonetheless, dog breeds are composed of related individuals across national boundaries. Therefore, it is important to take population structure and relatedness into account in models avoiding false positive associations. For this purpose we used a compressed linear model approach that has been proven useful in controlling for these effects in GWAS [31]. Concomitantly, this way reduced power for any mutation whose effects confounded with population structure. So, we may have missed contributory loci.

For eliminating potential associations due to population structure or relatedness we fitted the first three principal components derived from a pruned SNP set as covariates capturing false positive association due to population structure. Scatter plots for the first three principal components indicated only minor stratification due to sampling from the different European countries. Correction for multiple testing was done using 5142 SNPs which were considered as independent due to their correlation coefficients (r^2^<0.2). Using another less stringent threshold for moderate associations compensated for reduced power applying the compressed approach.

We identified one region significantly associated with DCM and five putative loci. Interestingly, we did not map DCM to regions harbouring known genes causing DCM in human. This fact might be explained that in human monogenic familial DCM cases were investigated. Here, causative mutations are rare in the population but have a strong effect on the phenotype. In Irish wolfhounds, DCM has a high incidence. Therefore, we looked for more common variants with smaller phenotypic effects. Our findings are supported by GWAS on ARCV in Boxers [19] and on DCM in Doberman Pinschers [22]. In the Boxer dog, the *striatin* gene seems to be causative for many ARVC cases in the United States. But the associated mutation explained not all cases and there are also unaffected dogs carrying the disease related mutation. Therefore, other still unknown genes are involved in developing ARCV. For DCM in Doberman Pinschers, one QTL on CFA5 was mapped. However, the best associated SNP identified about 50 percent of DCM cases in this breed. Further genomic regions were not mapped which supports the hypothesis that other loci with smaller effects contribute to the Doberman Pinscher DCM. Incidentally, it is worth to mention that the genomic region observed in Doberman Pinschers is not identical with any region mapped in Irish wolfhounds.

In Irish wolfhounds, the SNPs contributed in different degrees to the occurrence of DCM in Irish wolfhounds which could be shown by their odds ratios. Indeed, these loci were not able to explain all DCM cases in our cohort either due to incomplete linkage disequilibrium with the causative mutations or due to missing loci involved in DCM. There might be rare risk alleles or alleles with small effects which did not lead to significant peaks due to the limited numbers of diagnosed dogs. Due to inbreeding and to a genetic bottleneck by breed foundation, Irish wolfhounds share the longest linkage disequilibrium blocks observed in dog populations [32]. This fact facilitated identification of DCM associated regions but also impedes refinement of detected regions.

SNP BICF2G630134189 on CFA37 (g.31801266G>A) is the only marker which showed significant association with DCM in our study. It is located between *MOGAT1* and *ACSL3* two enzymes of the lipid synthesis. In rats, increased mRNA expression of *ACSL3* was observed in the progression of diabetic cardiomyopathy in the myocardium [33]. It was not shown if the elevated expression of *ACSL3* is cause or the result of the cardiomyopathy.

On CFA10, 15 and 21 the SNPs associated with DCM are located within genes. On CFA10, *ARHGAP8* (BICF2P801304, g.24159608A>C) is an interesting positional candidate gene. It is a member of the RhoA activating protein family which have been shown to regulate many aspects of intracellular actin dynamics. Actins are components of thin filaments of (cardiac) muscle cells and constituents of the cytoskeleton. Based on OR, BICF2P801304 on CFA10 might have the strongest effect on pathogenesis of DCM in Irish wolfhounds. However, the frequency of the minor allele of 0.06 in the Irish wolfhound population indicates only minor involvement in pathogenesis of DCM in Irish wolfhound population.

The positional candidate gene *FSTL5* (BICF2S23319191, g.61260406A>C) on CFA15 is proposed to be a prognostic marker for medullablastoma [34]. The gene is conserved in human, chimpanzee, dog, rat, chicken and zebrafish but a functional pathway has not been identified. On CFA21, *PDE3B* (BICF2P5199741, g.40670543G>A) is a cGMP inhibited phosphodiesterase. There is evidence that *PDE3B* is involved in regulation of lipolysis, lipogenesis, and insulin secretion [35]. For *ARGHAP*, *FSTL5* and *PDE3B* functional pathways need to be elucidated more detailed. Currently, we cannot evaluate if these genes might be involved in pathophysiology of cardiomyocytes or if one of proximate genes may have any impact on developing DCM in Irish wolfhounds.

On CFA1, SNP BICF2P937484 (g.123630555C>T) is associated with DCM suggesting that the nearest gene *TSHZ3* located 270 kb upstream may play a role in DCM in Irish wolfhounds. The gene is a zincfinger transcription factor which has been associated with respiratory rhythm and breathing control [36]. But, the gene may impact regulation in other pathways. On CFA17, four positional candidate genes GDAP2, WDR3, SPAG17 and TBX15 are located near SNP BICF2S23232515 (g.58604566C>T). These genes have not been connected to cardiovascular diseases. However, *HMGCS2*, 1.1 Mb away from SNP BICF2S23232515 encodes a mitochondrial enzyme of the metabolic pathway providing lipid derived energy for brain, heart and kidney in times of carbohydrate deprivation [37].

In total, we identified four positional candidate genes involved in lipid metabolism (*MOGAT1* and *ASCL3* on CFA37, *PDE3B* on CFA21 and *HMGCS2* on CFA 17). Until now, no causative mutation for DCM in genes of lipid metabolism has been reported. However, it is known that cardiac cells use mainly fatty acids as fuel. Between 60 to 90 percent of myocardial energy is supplied by them. There seem to be two pathways for lipid up-take either for VLDL-derived fatty acids or for chylomicron-derived fatty acids [38]. Generally, the uptake of fatty acids occurs either as lipoproteins or as free fatty acid associated with albumin. The key enzyme for breaking down the first ones is lipoprotein lipase (LPL). It was shown that over- or under-expression of *LPL* leads to deficient hearts in mice. Increased lipid up-take led to dilated cardiomyopathy [39]. Therefore, disturbances within pathways connected to or involved in lipid metabolism might play a role in developing DCM in Irish wolfhounds.

In summary, we identified one SNP significantly associated with DCM and five loci suggestively associated with DCM in Irish wolfhounds. The most interesting locus is located on CFA37. The risk allele is a common variant which might impair a big part of Irish wolfhound population.

## Materials and Methods

### Ethics Statement

All animal work has been conducted according to the national and international guidelines for animal welfare. The dogs in this study were included with consent of their owners. Data were collected during the routine veterinary cardiologic examination for DCM, diagnostic procedures which had to be carried out anyway. All blood-sampling of dogs was done in veterinary clinics for small animals by trained staff.

### Animals

In total, 190 samples from Germany, The Netherlands, Belgium, France and Sweden (the Swedish cohort includes dogs from several Scandinavian countries) were used for analysis (Table S1). There were 21 samples from France (19 cases and two controls) and five Scandinavian samples (one case and four controls). Mean age of diagnosis of all cases was 4.9 years. Dogs in the control cohort had to be DCM unaffected and 7 years old at diagnosis (mean age 7.7 years).

### Diagnosis of DCM

DCM affected dogs had to be diagnosed by veterinary cardiology specialists. The mean age of diagnosis was 4.9 years for all dogs. The diagnosis of DCM was based on the results of echocardiographic examinations. For diagnosing DCM in Irish wolfhounds echocardiographic criteria were left ventricular internal dimension at end-diameter systolic wider than 41 mm and wider than 61.2 mm at end-diastole, fractional shortening below 25%, and end-systolic volume indices greater than 41 ml/m^2^. Right ventricular dilatation was diagnosed when right ventricular internal dimensions, measured during end-diastole, were wider than 36.0 mm. Left or right atrial enlargement was present when the two-dimensional systolic internal diameter of the atrium measured parallel to the AV-valve, was wider than 56 mm [27]. Dogs in the control cohort had to be DCM unaffected and at least 7 years old at diagnosis (mean age 7.7 years).

### DNA Extraction

Genomic DNA was extracted from EDTA blood samples through a standard ethanol fractionation with concentrated sodiumchloride (6 M NaCl) and sodium dodecyl sulphate (10% SDS). Alternative, genomic DNA was extracted from buccal samples preserved in ethanol using the NucleoSpin 96 Tissue DNA Kit (NucleoSpin 96 Tissue DNA kit, Macherey Nagel, Hoerdt, France) according to the manufacturer’s instructions. Concentration of extracted DNA was determined using the Nanodrop ND-1000 (Peqlab Biotechnology, Erlangen, Germany). DNA concentration of samples for SNP chip analysis was adjusted to 70–120 ng/µl.

### Statistical Analysis

For genome-wide association analyses, DNA samples were genotyped on the canine high density bead chip (Illumina, San Diego, CA, USA) containing a total of 172,942 SNPs. Quality criteria were minor allele frequencies (MAF) >0.05, genotyping rate per SNP>0.90 and HWE test (p<0.00001). After filtering for quality criteria, 83,621 SNPs remained for analysis. The mean genotyping rate per individual was 0.995.

We performed a mixed model analysis (MMA) and a general linear model (GLM) analysis procedure using TASSEL version 3.088 [40]. The advantage of MMA or GLM over a simplistic approach without considering any other effects in modeling association can be seen in removing disturbing effects caused by data structure, different levels of relationships among animals and inbreeding. The model included the respective SNP genotypes, sex and inbreeding coefficients and the first three principal components as fixed effects and the genomic relationship matrix for the random genetic effect of the animal. For multiple testing we used a SNP set consisting of 5142 markers due to LD between SNPs, respectively. For representing linkage disequilibrium blocks we considered a window of 20 SNPs and shifted the window 5 SNPs forward in each step. One of a pair of SNPs was removed if the LD is greater than 0.2. The threshold of multiple tests was 9.72×10^−6^ at 0.05 of single test threshold. To obtain moderate evidence of association we used a less stringent threshold of 1×10^−4^.

For visualization and plotting of whole genome association, haplotype block analysis and haplotype association tests and results Haploview analysis was employed [41].

For allele frequencies of the SNPs, allele odds ratios, their corresponding CHI square and probability values the proc allele procedure of SAS, version 9.3 was carried out.

## Supporting Information

Figure S1 **Q-Q plot of general linear model using inbreeding coefficients, sex and the first three principal components as fixed effects.** The plot compares expected versus observed –log10 p-value for all 83,621 included in GWAS with the grey line corresponding to the null hypothesis of no association.
(TIF)

Figure S2 **Manhattan plot of genome-wide association study for dilated cardiomyopathy in Irish wolfhounds from Europe using a mixed model analysis.** X-axis indicates marker number. The genome-wide p-values (–log10 p-values) for the SNP effect are plotted against marker position on each chromosome. Chromosomes are differentiated by colours. Colours are given below the plot. Blue line indicates threshold value of probability for moderate association with DCM.
(TIF)

Figure S3 **Scatter plots of the first three principal components. A**: Principal component PC1 versus PC2 **B**: Principal component PC1 versus PC3 Turquoise triangels: Samples from Continental Europe (Germany, The Netherlands and Belgium) Blue diamonds: Scandinavian samples (Denmark, Norway and Sweden) Pink squares: samples from France
(TIF)

Figure S4 **Genome-wide association study in 190 European Irish Wolfhounds showed significant association for dilated cardiomyopathy on CFA1.** (**A**) SNPs and their corresponding –log10 p-values in a 1 Mb interval on dog chromosome 1 are shown (**B**). Gene annotation of the highest associated chromosomal region is shown (**C**). Gene annotation is based on dog genome assembly build 2.1. Some genes are still annotated as loc and numbers.
(TIF)

Figure S5 **Genome-wide association study in 190 European Irish Wolfhounds showed significant association for dilated cardiomyopathy on CFA10.** (**A**) SNPs and their corresponding –log10 p-values in a 0.5 Mb interval on dog chromosome 10 are shown (**B**). Gene annotation of the highest associated chromosomal region is shown (**C**). Gene annotation is based on dog genome assembly build 2.1. Some genes are still annotated as loc and numbers.
(TIF)

Figure S6 **Genome-wide association study in 190 Irish Wolfhounds from Europe showed significant association for dilated cardiomyopathy on CFA15.** (**A**) SNPs and their corresponding –log10 p-values in a 1 Mb interval on dog chromosome 15 are shown (**B**). Gene annotation of the highest associated chromosomal region is shown (**C**). Gene annotation is based on dog genome assembly build 2.1. Some genes are still annotated as loc and numbers.
(TIF)

Figure S7 **Genome-wide association study in 190 Irish Wolfhounds from Europe showed significant association for dilated cardiomyopathy on CFA17.** (**A**) Several SNPs in a 2 Mb interval on dog chromosome 17 were suggestively associated (**B**). Gene annotation of the highest associated chromosomal region is shown (**C**). Gene annotation is based on dog genome assembly build 2.1. Some genes are still annotated as loc and numbers.
(TIF)

Figure S8 **Genome-wide association study in 190 European Irish Wolfhounds showed significant association for dilated cardiomyopathy on CFA21.** (**A**) SNPs and their corresponding –log10 p-values in a 2 Mb interval on dog chromosome 21 are shown (**B**). Gene annotation of the highest associated chromosomal region is shown (**C**). Gene annotation is based on dog genome assembly build 2.1. Some genes are still annotated as loc and numbers.
(TIF)

Figure S9 **Genome-wide association study in 190 Irish Wolfhounds from Europe showed significant association for dilated cardiomyopathy on CFA37.** (**A**) SNPs and their corresponding –log10 p-values in a 3 Mb interval on dog chromosome 37 are shown. (**B**). Gene annotation of the highest associated chromosomal region is shown (**C**). Gene annotation is based on dog genome assembly build 2.1. Some genes are still annotated as loc and numbers.
(TIF)

Table S1 **Irish wolfhounds used for the genome-wide association study.** Distribution by diagnosis of dilative cardiomyopathy (DCM), sex, country of sampling and age at diagnosis or at last examination in years (AGE) is given.
(DOC)

Table S2 **Haplotype blocks associated to DCM in Irish wolfhounds.** Dog chromosome (CFA), haplotype block (bold letters are highest associated SNPs in GLM), bold, size of haplotype block in kb, frequency of haplotype block in all 190 Irish wolfhounds from Europe, frequency of haplotype block in affected and control dogs, CHI square value and corresponding p-value is given.
(DOC)
