# Supplementary figures and images for: Multiple Loci Are Associated with Dilated Cardiomyopathy in Irish Wolfhounds
Source: PLoS One. 2012 Jun 25;7(6):e36691. doi: 10.1371/journal.pone.0036691 (PMC3382626; doi:10.1371/journal.pone.0036691)

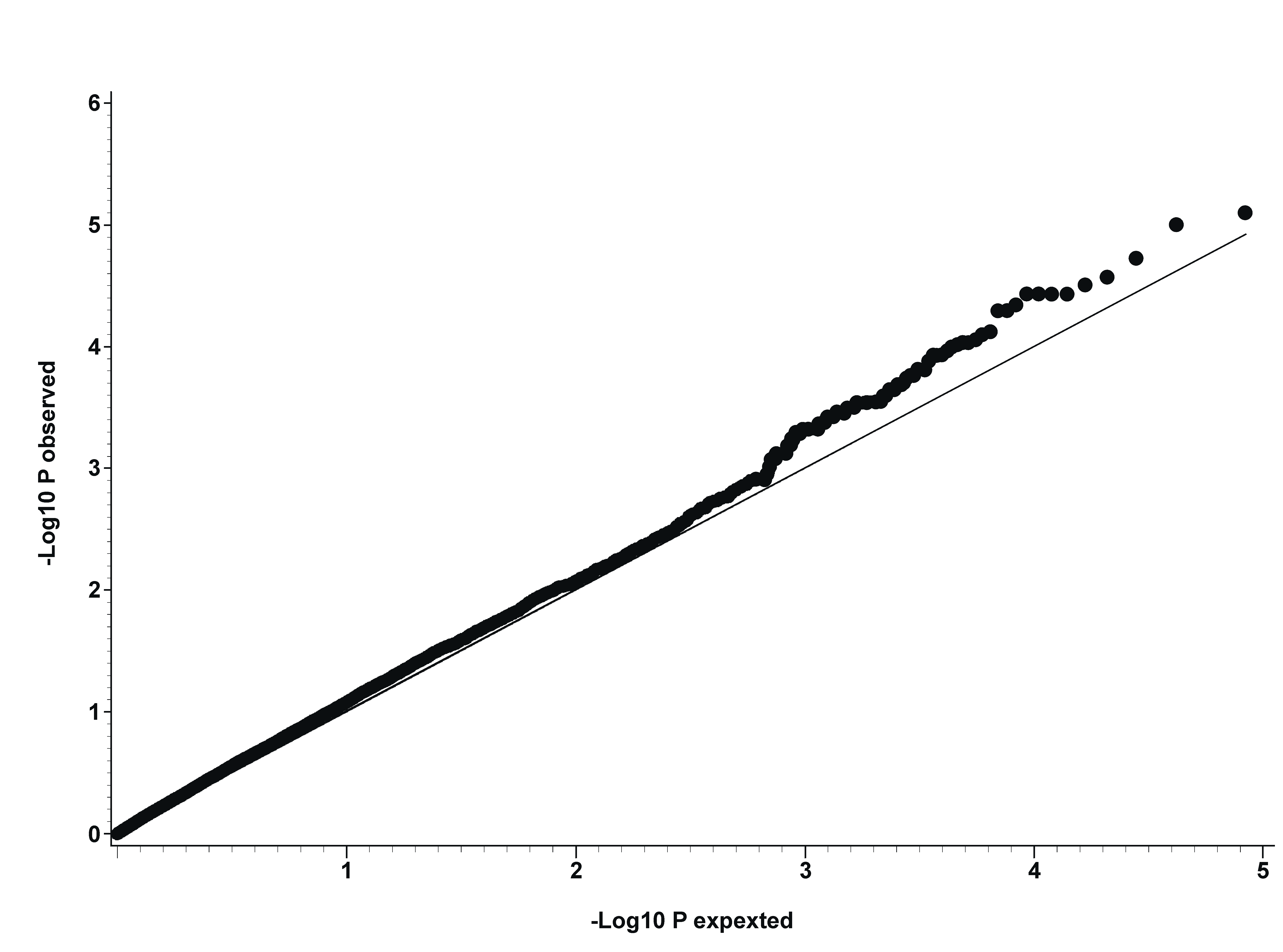

Supplement: Figure S1 — Q-Q plot of general linear model using inbreeding coefficients, sex and the first three principal components as fixed effects. The plot compares expected versus observed –log10 p-value for all 83,621 included in GWAS with the grey line corresponding to the null hypothesis of no association. (TIF) [file pone.0036691.s001.tif]

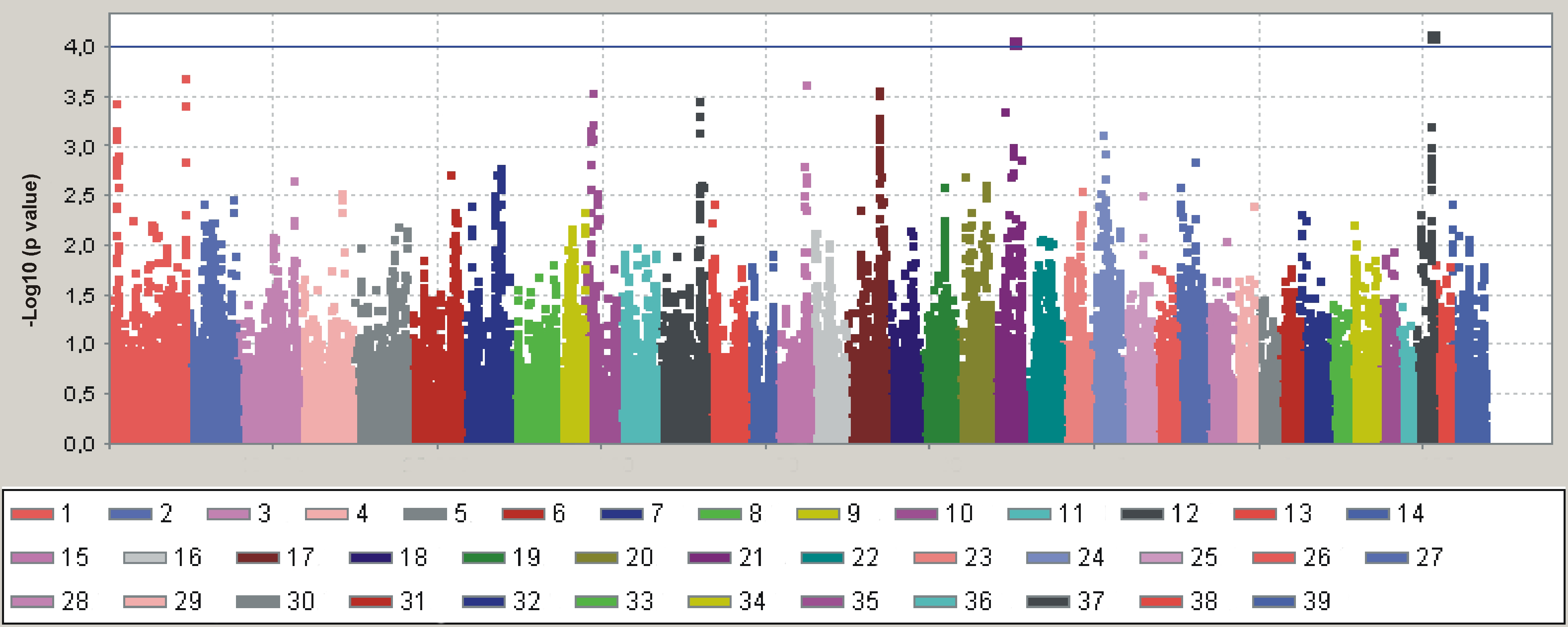

Supplement: Figure S2 — Manhattan plot of genome-wide association study for dilated cardiomyopathy in Irish wolfhounds from Europe using a mixed model analysis. X-axis indicates marker number. The genome-wide p-values (–log10 p-values) for the SNP effect are plotted against marker position on each chromosome. Chromosomes are differentiated by colours. Colours are given below the plot. Blue line indicates threshold value of probability for moderate association with DCM. (TIF) [file pone.0036691.s002.tif]

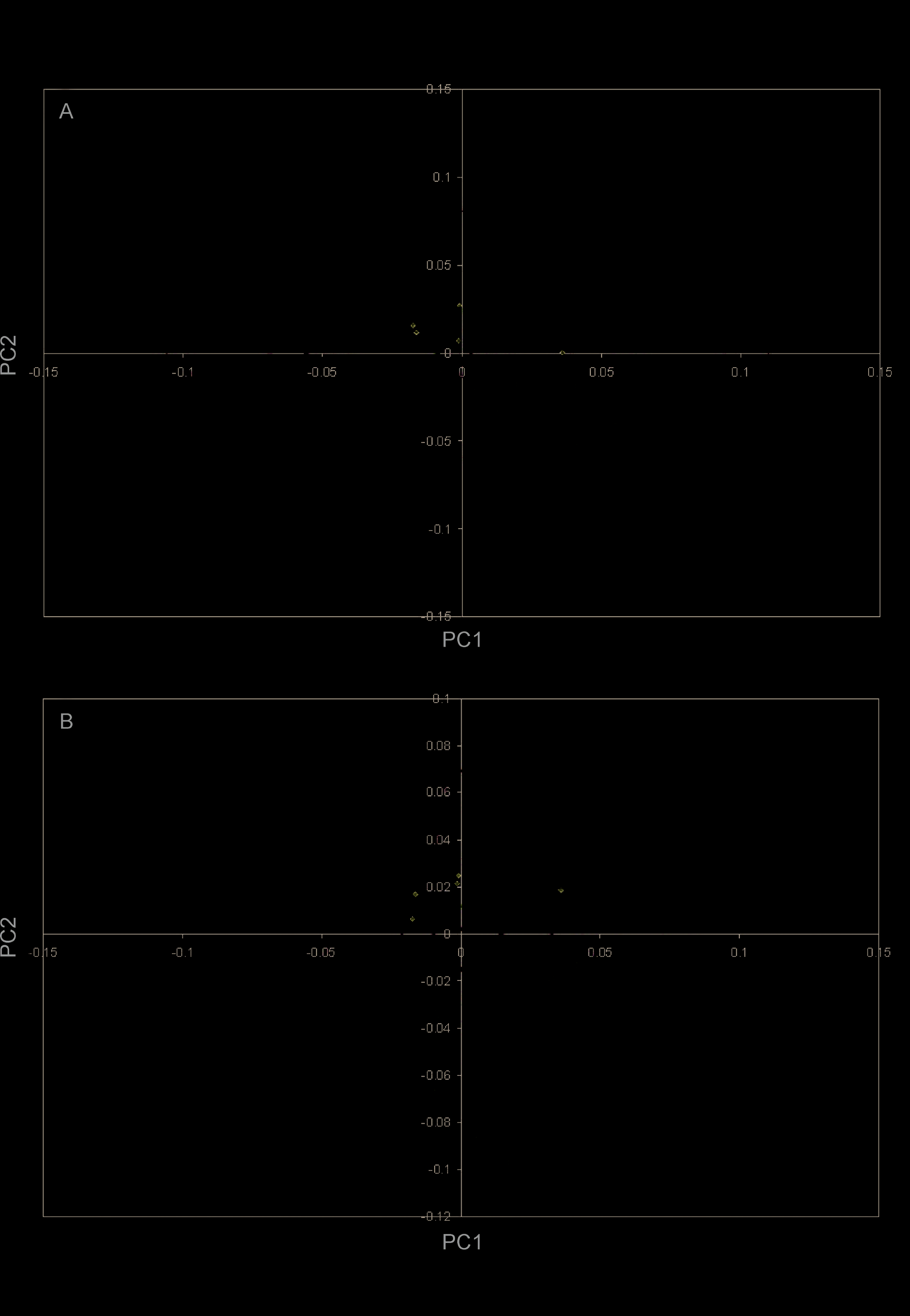

Supplement: Figure S3 — Scatter plots of the first three principal components. A: Principal component PC1 versus PC2 B: Principal component PC1 versus PC3 Turquoise triangels: Samples from Continental Europe (Germany, The Netherlands and Belgium) Blue diamonds: Scandinavian samples (Denmark, Norway and Sweden) Pink squares: samples from France (TIF) [file pone.0036691.s003.tif]

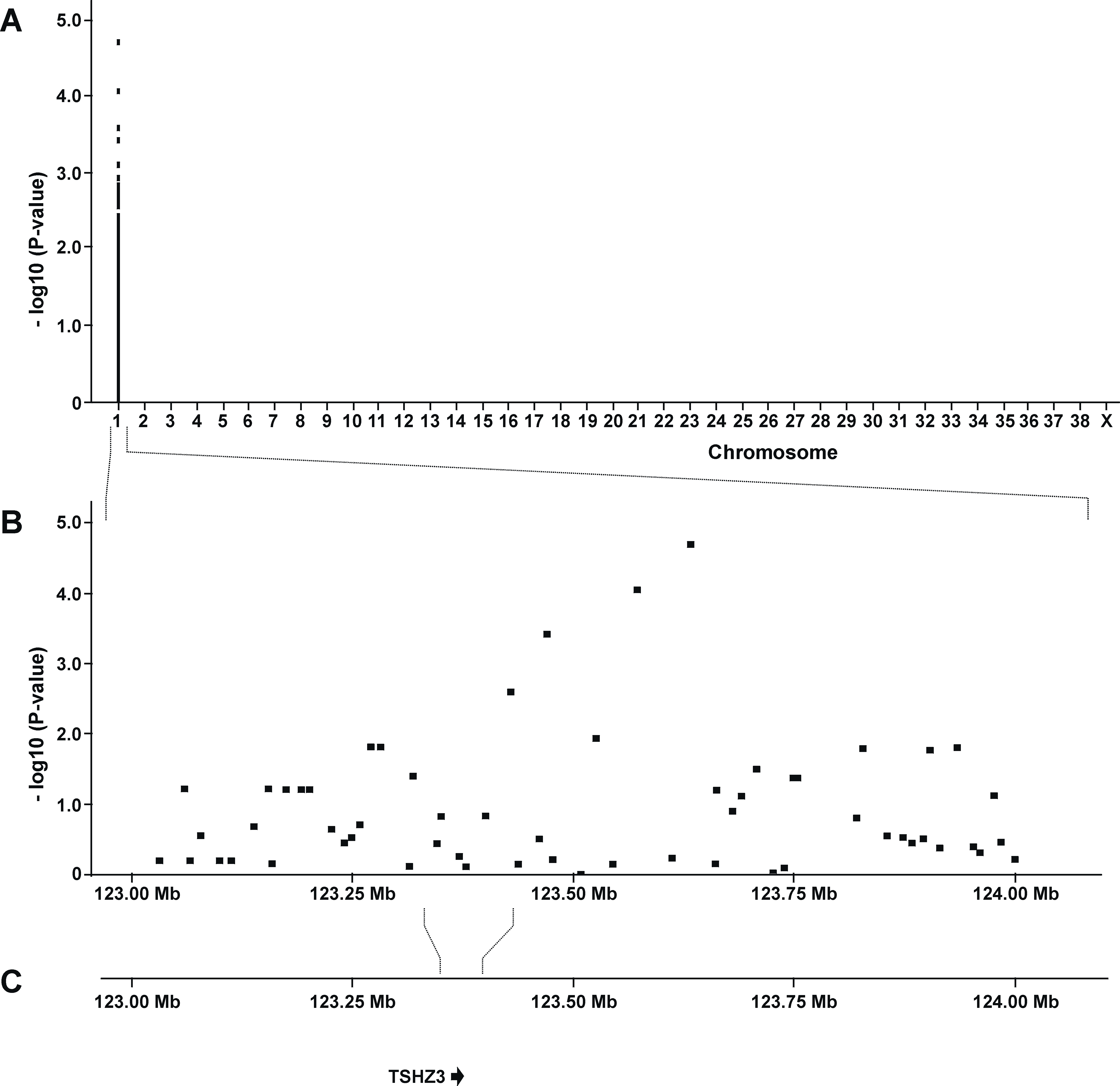

Supplement: Figure S4 — Genome-wide association study in 190 European Irish Wolfhounds showed significant association for dilated cardiomyopathy on CFA1. (A) SNPs and their corresponding –log10 p-values in a 1 Mb interval on dog chromosome 1 are shown (B). Gene annotation of the highest associated chromosomal region is shown (C). Gene annotation is based on dog genome assembly build 2.1. Some genes are still annotated as loc and numbers. (TIF) [file pone.0036691.s004.tif]

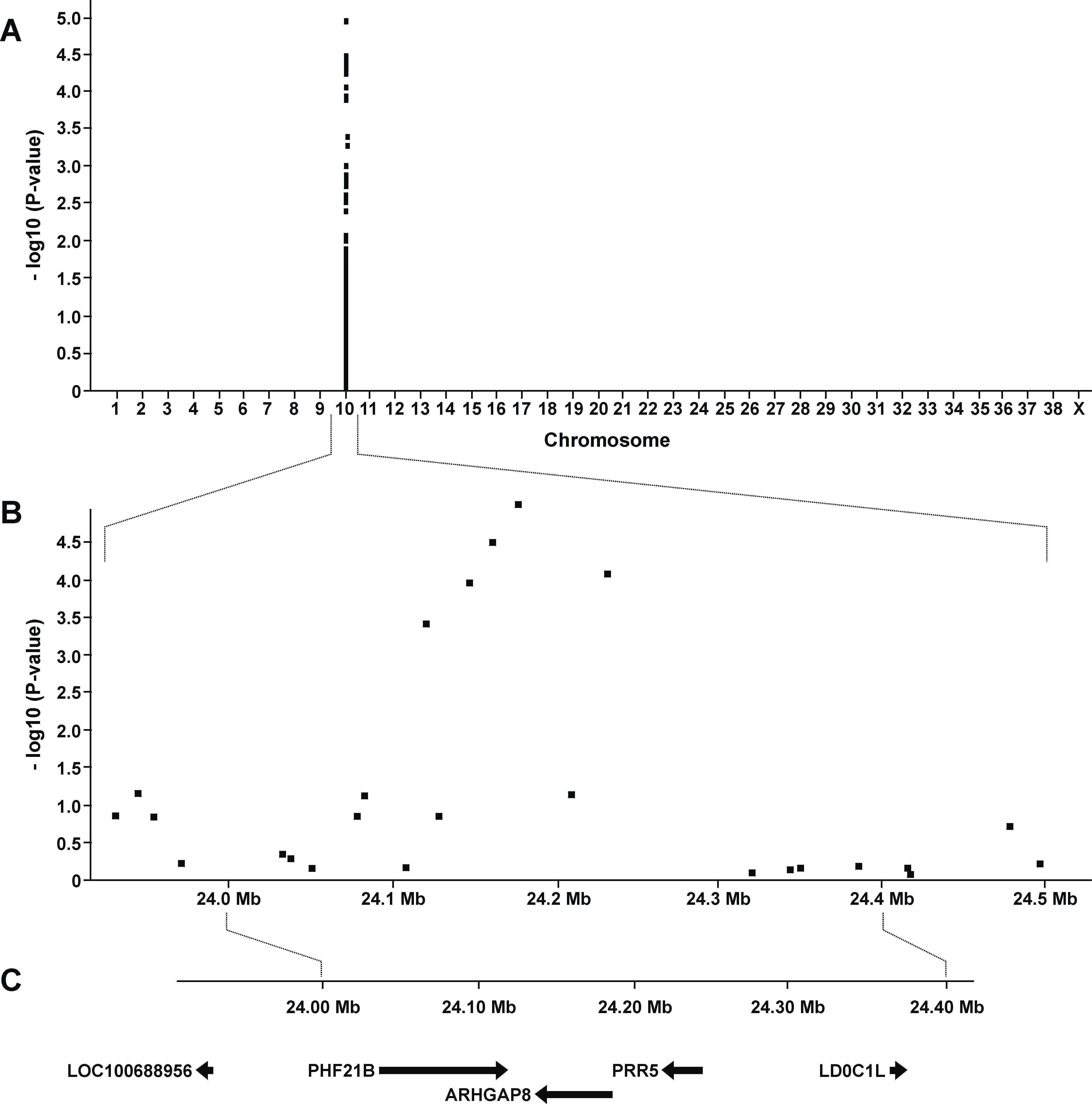

Supplement: Figure S5 — Genome-wide association study in 190 European Irish Wolfhounds showed significant association for dilated cardiomyopathy on CFA10. (A) SNPs and their corresponding –log10 p-values in a 0.5 Mb interval on dog chromosome 10 are shown (B). Gene annotation of the highest associated chromosomal region is shown (C). Gene annotation is based on dog genome assembly build 2.1. Some genes are still annotated as loc and numbers. (TIF) [file pone.0036691.s005.tif]

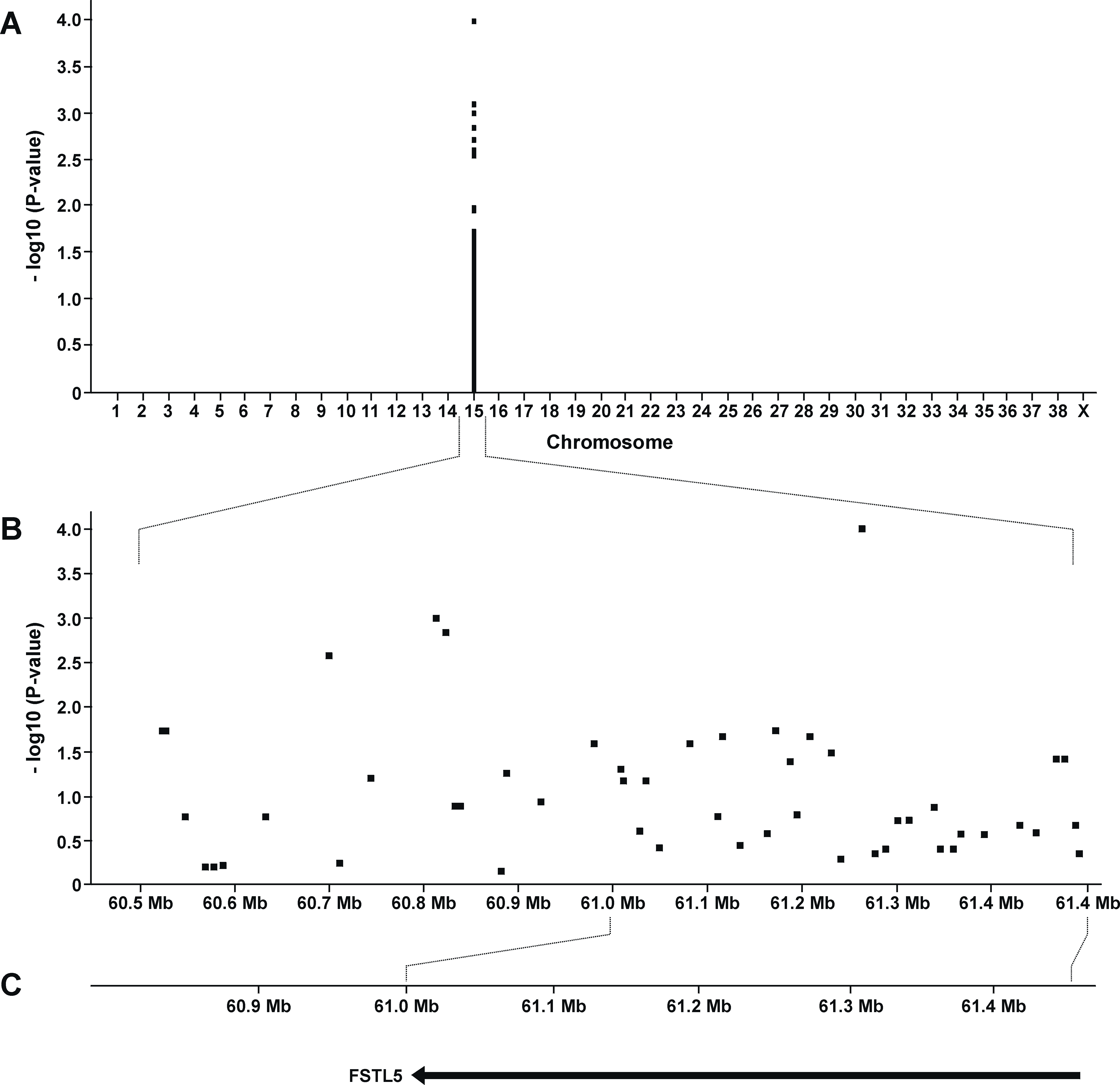

Supplement: Figure S6 — Genome-wide association study in 190 Irish Wolfhounds from Europe showed significant association for dilated cardiomyopathy on CFA15. (A) SNPs and their corresponding –log10 p-values in a 1 Mb interval on dog chromosome 15 are shown (B). Gene annotation of the highest associated chromosomal region is shown (C). Gene annotation is based on dog genome assembly build 2.1. Some genes are still annotated as loc and numbers. (TIF) [file pone.0036691.s006.tif]

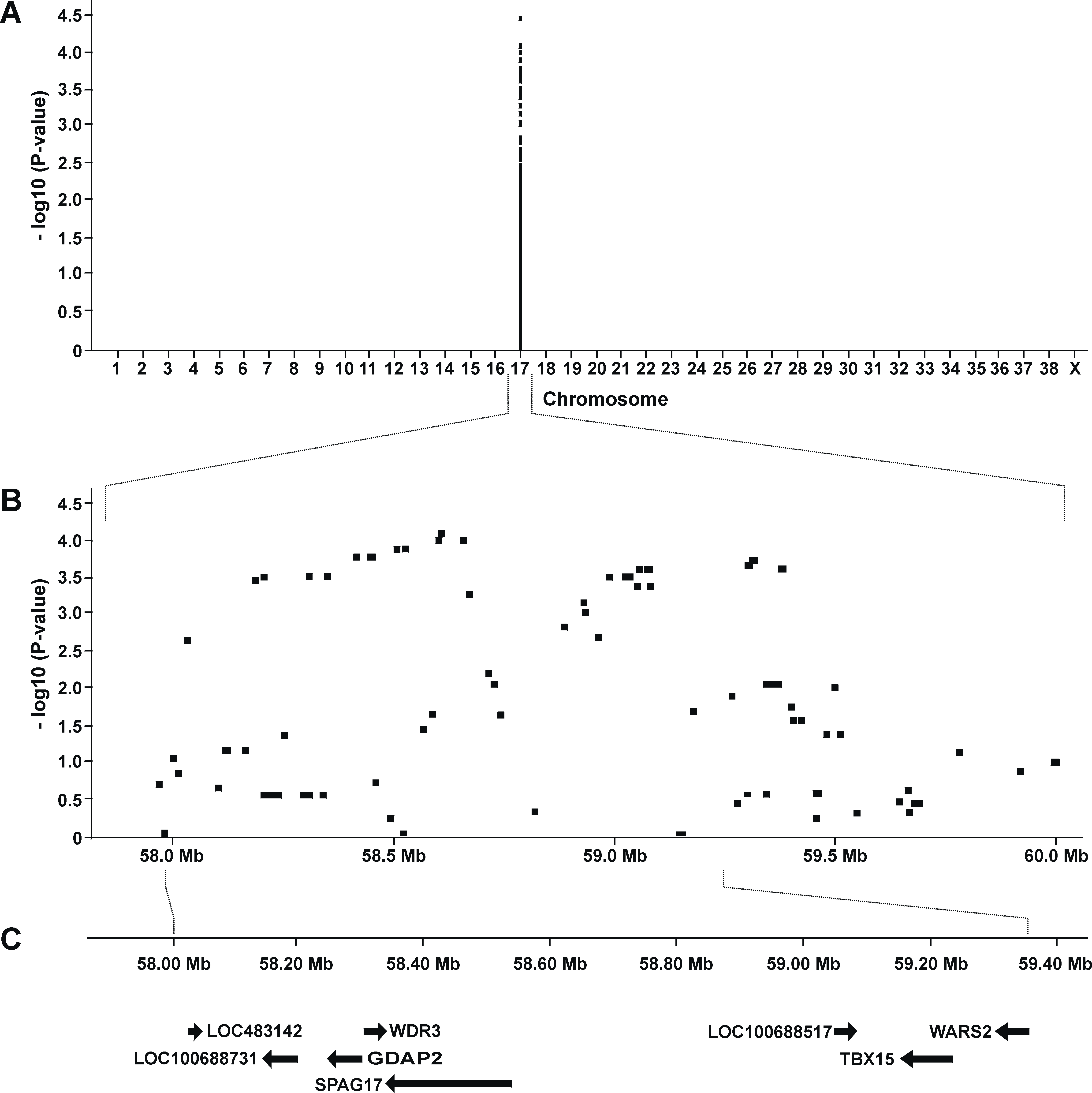

Supplement: Figure S7 — Genome-wide association study in 190 Irish Wolfhounds from Europe showed significant association for dilated cardiomyopathy on CFA17. (A) Several SNPs in a 2 Mb interval on dog chromosome 17 were suggestively associated (B). Gene annotation of the highest associated chromosomal region is shown (C). Gene annotation is based on dog genome assembly build 2.1. Some genes are still annotated as loc and numbers. (TIF) [file pone.0036691.s007.tif]

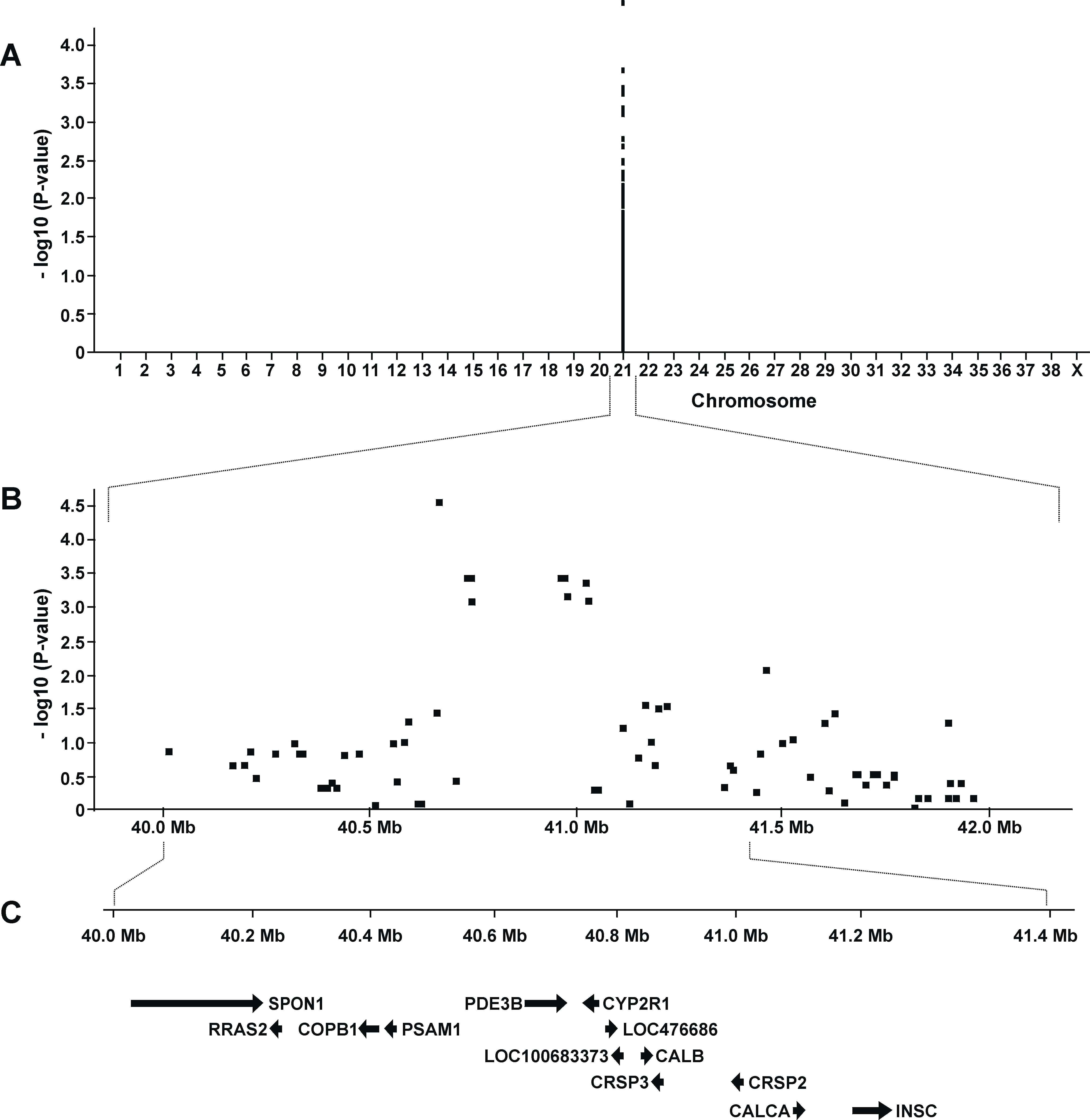

Supplement: Figure S8 — Genome-wide association study in 190 European Irish Wolfhounds showed significant association for dilated cardiomyopathy on CFA21. (A) SNPs and their corresponding –log10 p-values in a 2 Mb interval on dog chromosome 21 are shown (B). Gene annotation of the highest associated chromosomal region is shown (C). Gene annotation is based on dog genome assembly build 2.1. Some genes are still annotated as loc and numbers. (TIF) [file pone.0036691.s008.tif]

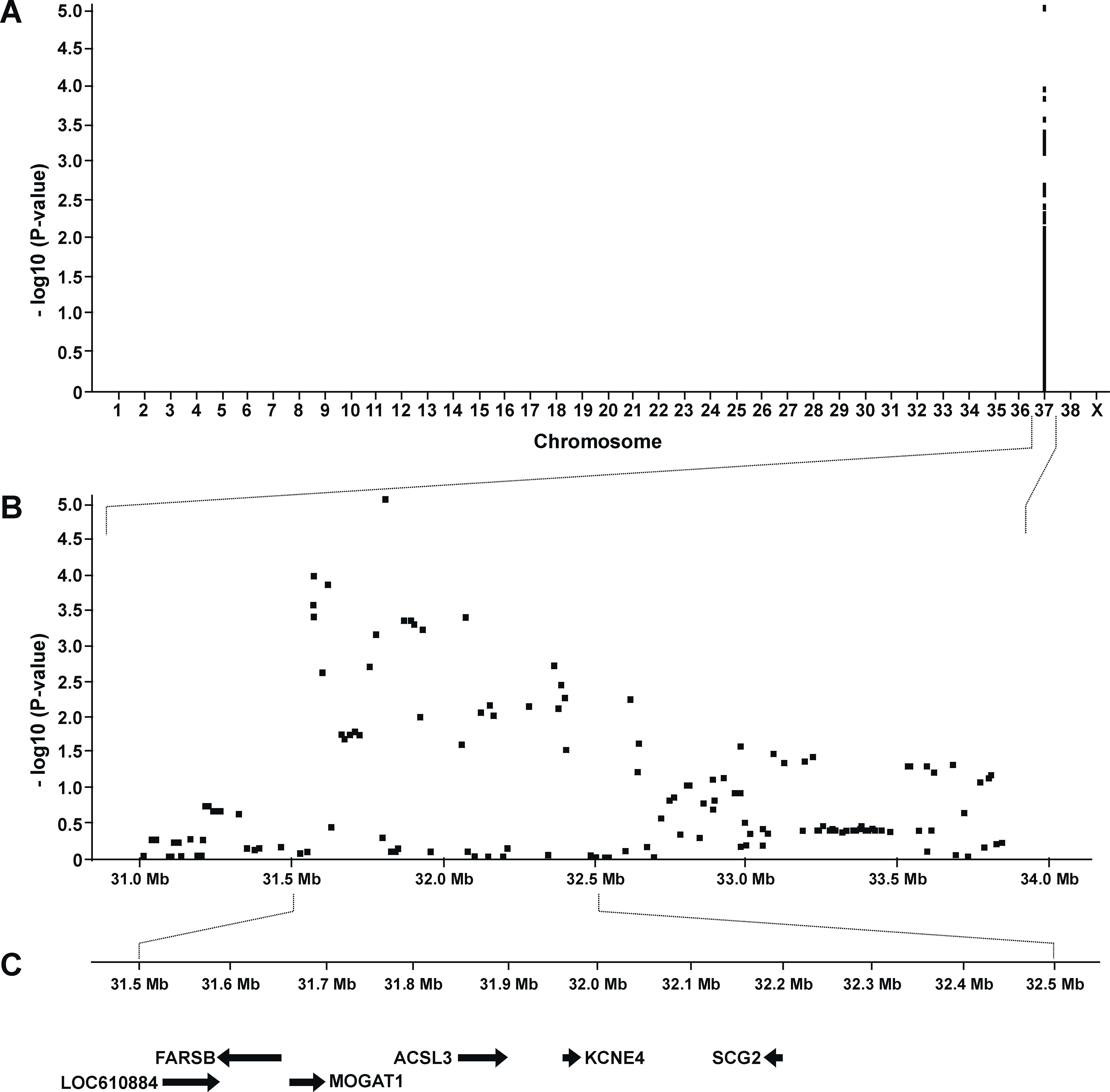

Supplement: Figure S9 — Genome-wide association study in 190 Irish Wolfhounds from Europe showed significant association for dilated cardiomyopathy on CFA37. (A) SNPs and their corresponding –log10 p-values in a 3 Mb interval on dog chromosome 37 are shown. (B). Gene annotation of the highest associated chromosomal region is shown (C). Gene annotation is based on dog genome assembly build 2.1. Some genes are still annotated as loc and numbers. (TIF) [file pone.0036691.s009.tif]
