# Supplementary material for: Multiple Loci Are Associated with Dilated Cardiomyopathy in Irish Wolfhounds
Source: PLoS One. 2012 Jun 25;7(6):e36691. doi: 10.1371/journal.pone.0036691 (PMC3382626; doi:10.1371/journal.pone.0036691)
Supplement: Table S1 — Irish wolfhounds used for the genome-wide association study. Distribution by diagnosis of dilative cardiomyopathy (DCM), sex, country of sampling and age at diagnosis or at last examination in years (AGE) is given. (DOC) [file pone.0036691.s010.doc]

| DCM status | Sex | | Country of sampling | | | AGE | Total |
| --- | --- | --- | --- | --- | --- | --- | --- |
|  | male | female | CE | FR | SW |  |  |
| Affected | 72 | 34 | 86 | 19 | 1 | 4.9 | 106 |
| Unaffected | 32 | 52 | 78 | 2 | 4 | 7.7 | 84 |
| Total | 104 | 86 | 164 | 21 | 5 | 6.3 | 190 |
