# Supplementary material for: Multiple Loci Are Associated with Dilated Cardiomyopathy in Irish Wolfhounds
Source: PLoS One. 2012 Jun 25;7(6):e36691. doi: 10.1371/journal.pone.0036691 (PMC3382626; doi:10.1371/journal.pone.0036691)
Supplement: Table S2 — Haplotype blocks associated to DCM in Irish wolfhounds. Dog chromosome (CFA), haplotype block (bold letters are highest associated SNPs in GLM), bold, size of haplotype block in kb, frequency of haplotype block in all 190 Irish wolfhounds from Europe, frequency of haplotype block in affected and control dogs, CHI square value and corresponding p-value is given. (DOC) [file pone.0036691.s011.doc]

| CFA | Haplotype block | Size (kb) | Frequency  haplotype | Frequency  affected dogs | Frequency  controls | Chi  square | P-  value |
| --- | --- | --- | --- | --- | --- | --- | --- |
| 1 | C-C-C-A-A-G-**C**-G-T-T | 204 | 0.418 | 0.464 | 0.356 | 4.39 | 0.036 |
|  | C-C-T-A-G-G-**T**-G-C-A |  | 0.124 | 0.082 | 0.181 | 8.45 | 0.0037 |
| 10 | G-C-T-**C-**T-G-T | 111 | 0.063 | 0.032 | 0.106 | 8.53 | 0.0035 |
| 15 | A-A-C-**A**-T-C | 78 | 0.258 | 0.218 | 0.312 | 4.306 | 0.038 |
| 17 | C-A-T-C-**C**-T-A-C-A-A-G-T-C-C-A-A-C-C-A-A-C-A-G-C-A-T-C-T-A-T-G-T | 794 | 0.266 | 0.200 | 0.356 | 11.589 | 7.0E-4 |
| 37 | G-A-C-T-T-T-G-A-**A**-A-A-C-C-A-G-A-T-C-T-C | 294 | 0.416 | 0.354 | 0.500 | 8.062 | 0.0045 |
|  | A-C-T-G-C-C-A-A-**G**-A-A-C-A-G-T-G-C-T-C-C |  | 0.331 | 0.400 | 0.237 | 11.044 | 9.0E-4 |
|  | G-A-C-T-T-C-A-A-**G**-A-A-C-A-G-T-G-C-T-C-C |  | 0.034 | 0.050 | 0.012 | 3.943 | 0.0471 |
